# PM_2.5_ affected ciliary beat frequency of axonemes via the cyclic AMP-dependent protein kinase a pathway

**DOI:** 10.3389/fpubh.2025.1529215

**Published:** 2025-04-25

**Authors:** Jinyan Pang, Zhiqin Xiong, Kexin Zhang, Yang Li

**Affiliations:** ^1^Department of Toxicology and Sanitary Chemistry, School of Public Health, Capital Medical University, Beijing, China; ^2^Laboratory for Clinical Medicine, Capital Medical University, Beijing, China

**Keywords:** PM2.5, ciliary beat frequency, cAMP, PKA, airway axonemes

## Abstract

Long-term inhalation of fine particulate matter (PM_2.5_) has been linked to the onset of various lung diseases. The mucociliary clearance system, acts as the primary host defense mechanism in the airways, with ciliary beat frequency (CBF) being a key parameter for assessing its functionality. The primary aim of this study was to demonstrate the impact of PM_2.5_ on CBF and to investigate the potential mechanisms by which PM_2.5_ induced changes in CBF through airway axonemes. Airway axonemes were extracted from bovine ciliated epithelium and treated with different concentrations of PM_2.5_*in vitro* for 10 min and 1 h to simulate short-term and prolonged exposures. Additionally, the pathway was examined using PKA activator (cAMP) and PKA inhibitor (PKI) on ciliary axonemes. The results revealed that PM_2.5_ stimulated CBF in airway axonemes via the cAMP-PKA pathway. Low concentrations and short-term exposure to PM_2.5_ stimulated CBF elevation, however, high concentration and prolonged exposure to PM_2.5_ might damage respiratory cilia, thereby increasing the risk of respiratory diseases.

## Introduction

Evidence indicated that air pollution had already become a significant health threat to global public health at this stage. According to statistics from the World Health Organization, non-communicable diseases caused by environmental and household air pollution, including respiratory diseases, cardiovascular and cerebrovascular diseases, and cancer, resulted in approximately 7 million deaths annually (1). Moreover, in the analysis of specific risk factors for the global disease burden in 2021, particulate matter air pollution was the largest contributor to the global burden of diseases, accounting for 8.0% (95% UI 6.7–9.4%) of the total disability-adjusted life years (DALYs) (2). Statistical data indicated that in China, approximately 350,000 to 500,000 people died annually due to air pollution (3). Fine particulate matter (PM_2.5_) is airborne particles with a diameter ≤ 2.5 μm. It was one of the most complex and most harmful in the air contaminants (4). Environmental PM_2.5_ is composed of various components, with different components exhibiting varying degrees of toxicity to humans. Moreover, PM_2.5_ particles were easily inhaled and deposited in the airways, causing lung injury and exacerbating a range of respiratory diseases, such as chronic obstructive pulmonary disease (5), pulmonary fibrosis (6), asthma (7), and cancer (8).

To combat the threat posed by fine particulates, pathogens, and other harmful substances, the body has strategically positioned the mucociliary clearance (MCC) system as the initial line of defense within the respiratory tract. The MCC system is the primary defense mechanism of the respiratory epithelium. The mucus, secreted by goblet cells within the epithelium and submucosal glands, serves as a carrier, capturing and transporting these particles. Cilia propel the mucus and the particles it adsorbs from the bronchi toward the gastrointestinal tract through coordinated beating patterns, thereby clearing inhaled particles, maintaining airway patency, and preventing particles from penetrating deeper into the lungs (9). Ciliary beat frequency (CBF) is an important parameter of MCC efficiency (10), and CBF has been proved to be an important physiological marker of airway mucosal health (11). When ciliary function was impaired, it could lead to upper respiratory tract inflammation, which might be chronic, recurrent, and more severe, resulting in dysfunction of MCC (12). CBF was influenced by various factors, in addition to genetic causes of ciliary dyskinesia, including temperature (13), pH value (14), tobacco (15) and alcohol (16). Some medications used to treat asthma or other respiratory conditions, such as *α*-sympathomimetic drugs (17), carbocysteine (18), naringenin (19), and long-acting muscarinic antagonists (20), have also been shown to affect CBF. In addition, it has been reported that PM_2.5_ entering the respiratory tract could cause structural and functional abnormalities in airway cilia, leading to mucus transport failure, which is one of the reasons for the development of respiratory diseases (21). However, the role of environmental pollutant PM_2.5_ in the beating of airway-ciliated epithelial cell axonemes has rarely been described in studies, and the underlying mechanisms remain unclear.

CBF of the ciliary axonemes is regulated by a variety of intracellular physiological molecules. Cyclic guanosine monophosphate (cGMP), cyclic adenosine monophosphate (cAMP) and Ca^2+^ are considered to be important secondary messengers mediating changes in CBF (22). The main cellular receptor for the second messenger cAMP is cAMP-dependent protein kinase A (PKA). The cAMP-PKA signaling is one of the common regulatory pathways in mammals. It has been previously demonstrated that ATPase-dependent motility of cilia in bovine bronchial epithelial cells was regulated by cyclic nucleotides, and that cAMP regulated ciliary CBF via PKA (23). The cAMP-PKA pathway was considered to play a crucial role in the key functions of MCC and airway defense. Previous studies had demonstrated that the activation or inhibition of the cAMP-PKA pathway could directly influence the development of several respiratory diseases, including asthma, airway fibrosis, chronic obstructive pulmonary disease (COPD), and cancer (24–26). The previous sections have adequately elucidated that the cAMP-PKA signaling pathway in the respiratory tract was essential for the efficient clearance of particulate matter and airway defense. However, it is not yet fully understood whether PM_2.5_ can impair ciliary motility by affecting the cAMP-PKA pathway.

The aim of the study was to investigate the effects of PM_2.5_ on CBF in respiratory ciliary axonemes and the underlying mechanisms. The study was divided into two parts. In the first part, CBF of airway ciliary axonemes cultured *in vitro* was measured following exposure to different concentrations of PM_2.5_ for 10 min and 1 h, respectively, to observe the impact of varying exposure times and concentrations of PM_2.5_ on axoneme motility. In the second part, the axonemes were exposed to different concentrations of PM_2.5_ under the influence of cAMP (a PKA activator) and PKI (a PKA inhibitor). This was done to study the effects of PM_2.5_ on the cAMP-PKA pathway, which is involved in respiratory mucosal cilia beating, thereby elucidating the mechanisms involved.

## Methods

### Collection and characterization analysis of PM_2.5_ sample

The collection and extraction of PM_2.5_ had been conducted in our previous study (27). In summary, PM_2.5_ was collected through a quartz fiber filter (8 × 10 in, Pall, USA) with an airborne particulate sampler (TH-1000CII, Wuhan Tianhong, China) at a flow rate of 10^5^ m^3^/min for 24 h. The fine particles were extracted from the filter using ultrasonication and then concentrated through vacuum freeze-drying. The obtained product was weighed and stored at a temperature of −20°C. The PM_2.5_ was resuspended in deionized water to reach the requisite concentrations for the experiment and stored at 4°C. Simultaneously, the PM_2.5_ resuspension was monitored using a transmission electron microscope (TEM) (JEOL JEM2100, Japan) to examine the morphology of PM_2.5_.

### Extraction and preparation of airway axonemes

The isolation of bovine respiratory ciliary axonemes had been conducted using a method previously described (28). Initially, fresh bovine bronchial tracts were collected from a local abattoir and rinsed twice with phosphate-buffered saline (PBS) before the removal of excess fat and connective tissue. Subsequently, 15 mL of extraction buffer, comprising 1 mM EDTA, 20 mM Tris–HCl, 10 mM calcium chloride, 50 mM NaCl, 1 mM dithiothreitol, 100 mM Triton X-100, and7 mM 2-mercaptoethanol, was added. Large hemostats were then used to close both the proximal and distal ends of the trachea. Following vigorous oscillation of the trachea in both upward and downward directions for 90 s, the extraction buffer with the released axonemal proteins was passed through a 100 μm polypropylene mesh. The prepared sample was subsequently centrifuged at 17,250 g for 7 min, after which the supernatant was discarded and the axonemal protein pellet was resuspended in a resuspension buffer at a concentration of 1 mg/mL. The resuspension buffer contained 50 mM KCl, 20 mM Tris HCL, 0.5 mM EDTA, 4 mM MgCl_2_, 10 mM soybean trypsin inhibitor, 1 mM dithiothreitol, and 25% sucrose (w/v). The isolated axonemes were stored at −80°C in a refrigerator for 6 months.

### Experimental treatment of airway axonemes

Frozen aliquots of axonemes were thawed on ice, kept at 4°C, and gently aspirated with a pipette to minimize aggregation for subsequent use. A sonicator (Branson 2,510, Branson Ultrasonics, United States) was used to prepare PM_2.5_ suspensions by treating for 5 min at 160 W and 20 kHz. The PM_2.5_ suspensions were then diluted to five concentrations (6.25, 12.5, 25, 50, and 100 μg/mL). To activate or inhibit the airway axonemes, corresponding concentrations of 10 μM cAMP and 2 μg/mL PKI were added, respectively. Finally, axoneme samples were added and diluted to a final concentration of 0.25–0.5 mg/mL. The mixtures were then incubated in microcentrifuge tubes at room temperature for 10 min or 1 h. At each time point measured, 10 μL drop was removed from the sample microcentrifuge tube and placed into one well of a 48-well polystyrene tissue culture plate, breaking the meniscus of the drop so that the sample was evenly distributed. Axonemes maintained in a medium without PM_2.5_ served as the control. Each experimental group comprised five replicate wells.

### Measurement of CBF

The CBF of isolated axonemes was determined using the SAVA system to quantify the impact of PM_2.5_ on ciliary beating. Axonemes were fixed by centrifugation to prevent movement, and the majority of axonemes exhibited flexure in each field of view. As previously described, the experiments were conducted with temperature control using a thermostatic stage, maintained at 23°C ± 0.5°C. Motility was recorded for 10 min at a sampling rate of 85 frames per second, for all experimental groups. Axonemes exhibiting a frequency of ≤ 2 Hz were deemed nonmotile and thus excluded from the analysis. Similarly, data points exhibiting a frequency of less than 10% of the original number of beats were excluded from the analysis. For each experimental condition, at least six separate fields were captured, analyzed, and expressed as the mean ± standard deviation per data point.

### Statistical analysis

A minimum of three separate experiments were performed for each unique parameter. The experimental data were analyzed using GraphPad Prism 9.0 software and are presented as mean ± S.D. For data comparisons involving three or more groups used one-way analysis of variance (one-way ANOVA) with Dunnett’s test, and Student’s t-test was used for comparisons between two groups. *p* < 0.05 was considered to be statistically significant.

## Results

### Characterization analysis of PM_2.5_ samples

**Figure 1:**
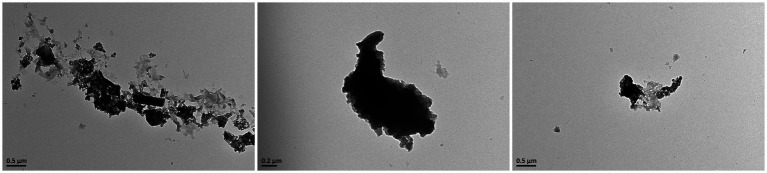
Upon observation with transmission electron microscopy, it was discovered that PM_2.5_ consists of particles with irregular shapes, which are either dispersed individually or aggregated into clusters.

The morphological characteristics of PM_2.5_ were observed and photographed using TEM. As shown in Figure 1, after the fine particulate matter sample, preserved by freeze-drying, was resuspended in deionized water. PM_2.5_ particles displayed a range of sizes and shapes, both as individual particles (monomers) and as aggregates, predominantly consisting of irregular fine and ultrafine particles.

### PM_2.5_ affected the CBF of ciliary axonemes

**Figure 2:**
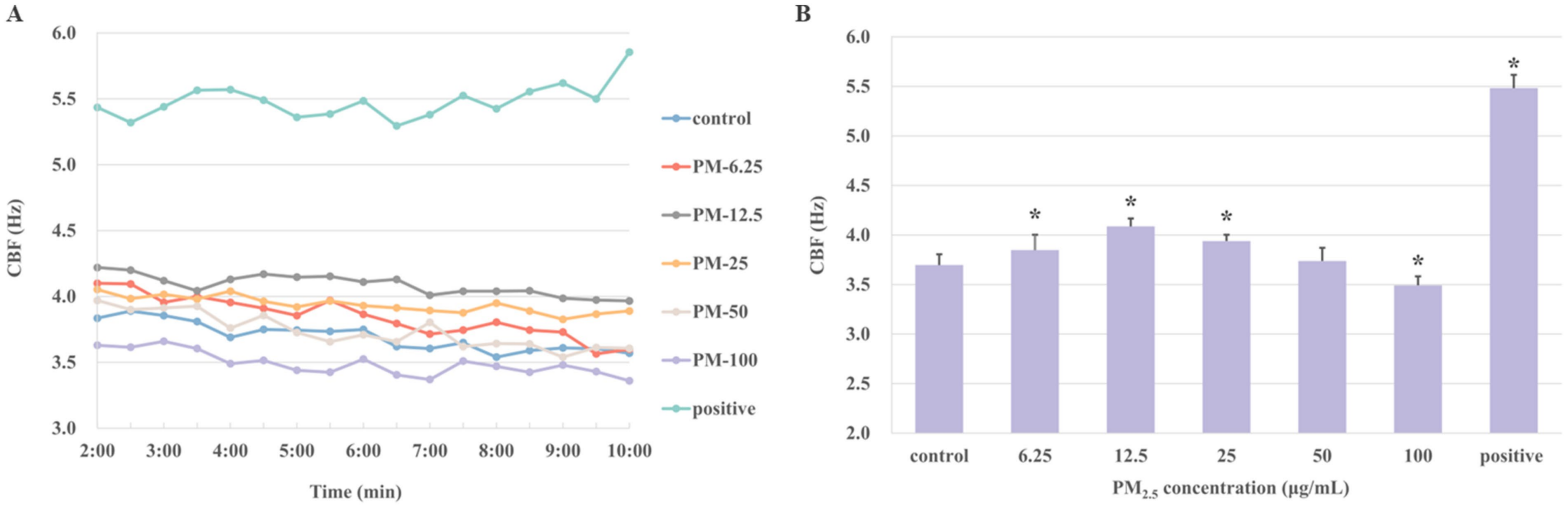
The CBF was detected after the ciliary axonemes were exposed to varying concentrations of PM_2.5_ (6.25, 12.5, 25, 50, 100 μg/mL) for 10 min. Ciliary axons exposed to 10 μM cAMP was set as positive control. **(A)** Ciliary axons were exposed to different concentrations of PM_2.5_ for 10 min. Given the necessity for system stabilization, CBF was monitored from 2 min onwards at 30s intervals. **(B)** The averages of CBF at different concentrations of PM_2.5_. The findings indicated that axonemes increased CBF when treated with low concentrations of PM_2.5_, particularly at 12.5 and 25 μg/mL PM_2.5_ concentrations, and ciliary beating was inhibited at high concentration (100 μg/mL). The data were expressed as mean ± S.D. of three independent experiments. *p < 0.05 PM_2.5_-treated group compared with the control group.

In order to evaluate the possible toxic effects of PM_2.5_ on ciliary axonemes, the changes in CBF were determined after treating the axonemes with various PM_2.5_ concentrations (6.25, 12.5, 25, 50, and 100 μg/mL) for 10 min, respectively. Results in Figure 2A showed the change of CBF after 10 min of treating with different concentrations of PM_2.5_. Figure 2B showed the average value of CBF over a 10-min period. In the control group, the average CBF was 3.75 ± 0.10 Hz. With the concentrations of PM_2.5_ increased, the average CBF reached the highest at 12.5 μg/mL, which was 4.09 ± 0.08 Hz and approximately 10% higher than the control group (*p* < 0.05). Nevertheless, when the concentration was up to 100 μg/mL, the CBF of ciliary axonemes decreased to 3.49 ± 0.09 Hz, representing a decrease of about 6.9% compared to the control group (*p* < 0.05). The results suggested that exposure to low concentrations (6.25, 12.5, 25 μg/mL) of PM_2.5_ might promote the frequency of axoneme beating and that a decrease in CBF might occur when the axonemes were exposed to high-concentration PM_2.5_.

### The effect of PKA inhibitor on the axonemes exposed to PM_2.5_

**Figure 3:**
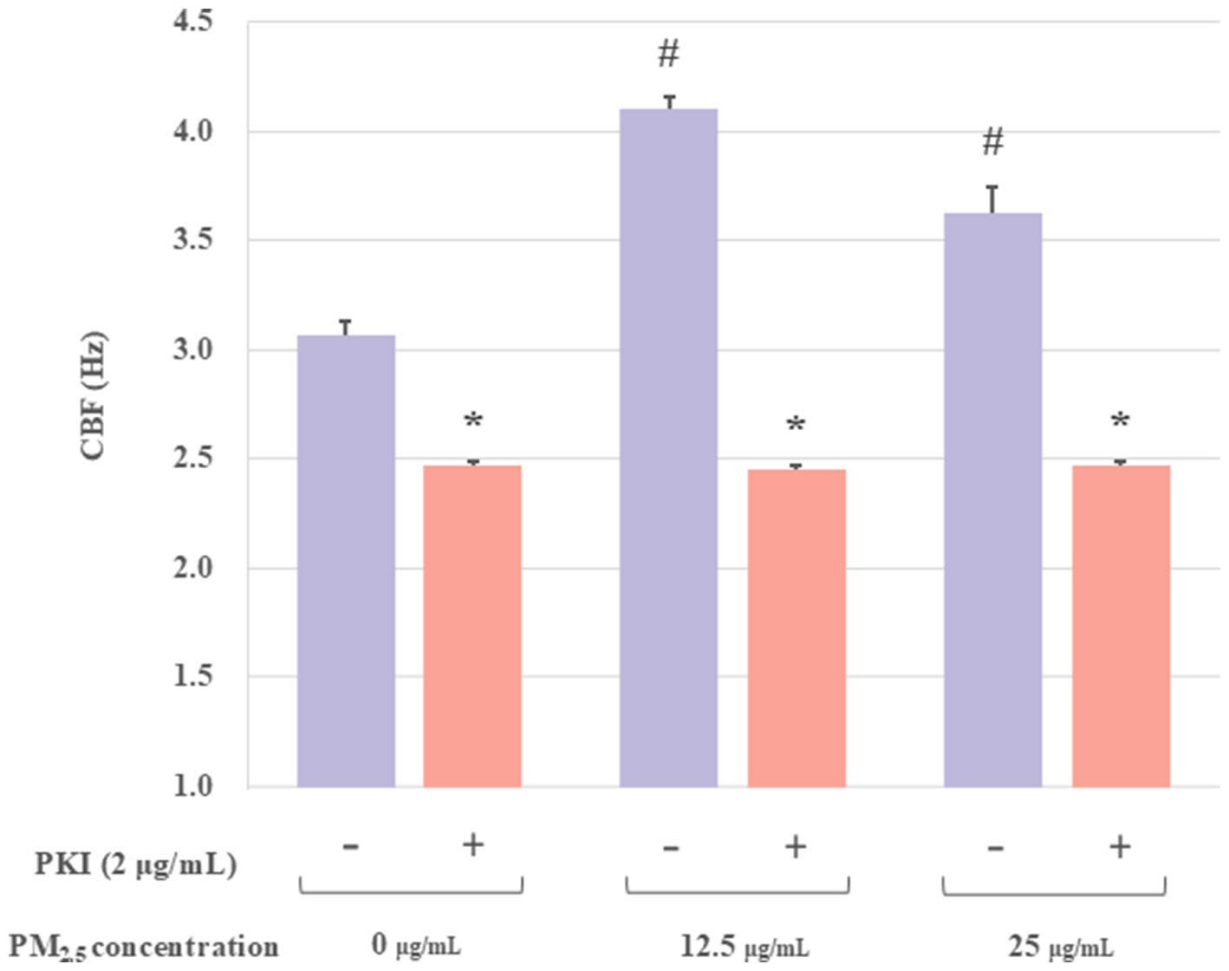
The effect of PKI on the axonemes exposed to PM_2.5_. The results indicated that the PKA inhibitor (PKI) significantly inhibited PM_2.5_-treated CBF. All experiments were repeated three times. *p < 0.05, PM_2.5_-treated group compared with corresponding PM_2.5_ plus PKI group. #*p* < 0.05, PM_2.5_-treated group without PKI compared with the control group.

Previous studies demonstrated that the stimulation of PKA resulted in the phosphorylation of downstream target proteins, thereby stimulating CBF. Concurrently, PKA played a dual role in ciliary muscle regulation, as it potently activated ciliary muscle pulsation by releasing Ca^2+^ from intracellular stores and moderately prolonged CBF through a Ca^2+^-independent mechanism (29). Protein kinase inhibitor peptide (PKI) was an endogenous thermostable peptide that effectively and specifically inhibited the activity of the free catalytic subunit of cAMP-dependent protein kinase (30). To further investigate whether PKA mediates PM_2.5_-induced CBF alterations, the PKA inhibiter PKI was introduced to this study. If PKI effectively inhibits PM_2.5_-induced ciliary CBF elevation, this would indicate that PKA activation plays a critical role in PM_2.5_-mediated ciliary motility increases. Thus, the groups were set as: control group, control plus PKI-treated group, PM_2.5_-treated groups (12.5 or 25 μg/mL), and PM_2.5_ (12.5 or 25 μg/mL) plus PKI-treated groups. The PM_2.5_ plus PKI-treated group showed a significant decrease compared with the PM_2.5_-treated group (Figure 3). At a PM_2.5_ concentration of 12.5 μg/mL, the mean CBF was 4.12 ± 0.12 Hz. However, the CBF of the PM_2.5_ concentration of 12.5 μg/mL plus PKI group was 2.46 ± 0.03 Hz, which was approximately 1.71 times lower than that of the PM_2.5_-treated group. Moreover, the CBF in both PM_2.5_-treated groups was found to be significantly different from that of the control group. However, no difference was observed between the PM_2.5_ plus PKI-treated groups and the control plus PKI-treated group. These results suggested that PKI could effectively inhibit PM_2.5_-induced changes in CBF. Thus, PM_2.5_ might affect the CBF of ciliary axons by modulating the activity of PKA.

### The effect of cAMP on the axonemes exposed to PM_2.5_

**Figure 4:**
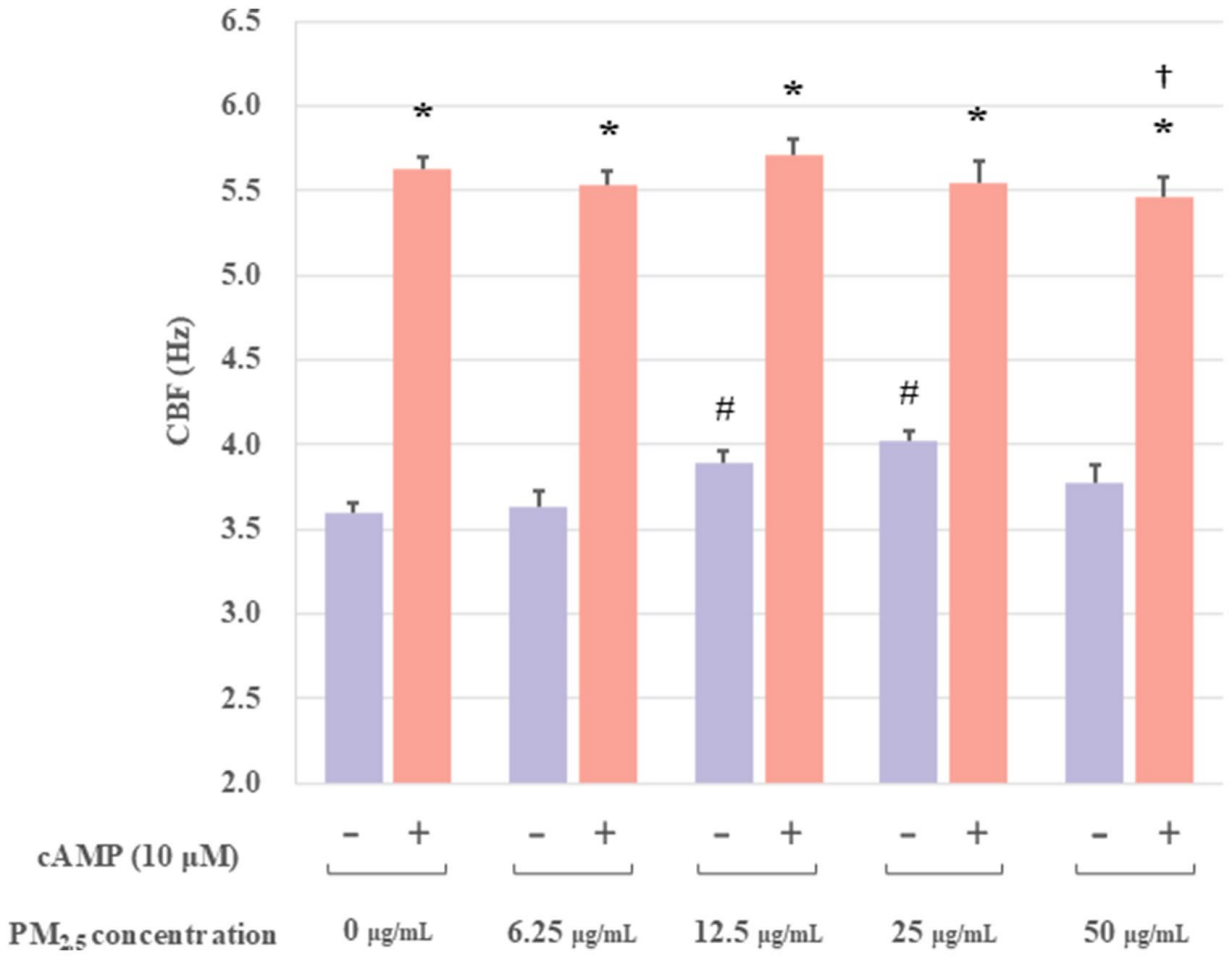
The effect of cAMP on the axonemes exposed to PM_2.5_. These results indicated that cAMP significantly boosted ciliary axon activity when exposed to PM_2.5_. In addition, the 50 μg/mL PM_2.5_ plus cAMP-treated group resulted in significantly lower CBF compared to the 25 μg/mL PM_2.5_ plus cAMP-treated group. *p < 0.05 PM_2.5_ plus cAMP-treated group compared to PM_2.5_-treated group. †*p* < 0.05 PM_2.5_ plus cAMP-treated group compared to the control plus cAMP treated group. #*p* < 0.05 PM_2.5_-treated group compared to the control group.

PKA, the protein kinase of cAMP, could be specifically activated by cAMP. Furthermore, it had been demonstrated that increases in CBF were associated with cAMP activation. To determine whether the increase in CBF stimulated by PM_2.5_ was related to the activation of cAMP, extracted axonemes were exposed to varying concentrations of PM_2.5_ (6.25, 12.5, 25 and 50 μg/mL) for 10 min, followed by incubation in the presence or absence of 10 μM cAMP, and then measured the CBF. The experimental groups were set up as follows: a control group, a control plus cAMP-treated group, PM_2.5_-treated (6.25, 12.5, 25, and 50 μg/mL) group, and PM_2.5_ (6.25, 12.5, 25, and 50 μg/mL) plus cAMP-treated group. As illustrated in Figure 4, the PM_2.5_ plus cAMP-treated group exhibited significantly higher CBF than the PM_2.5_-treated group (*p* < 0.05). This result suggested that cAMP could markedly enhance the activity of ciliary axons under PM_2.5_ treatment. Moreover, at PM_2.5_ concentration of up to 50 μg/mL, a significant downward trend in CBF was observed compared to the control plus cAMP-treated group (*p* < 0.05). The results suggested that high concentrations of PM_2.5_ might reduce PM_2.5_-induced axonal beating by affecting the activation of cAMP, thereby inhibiting PKA activity.

### The effect of long-term PM_2.5_ exposure on ciliary axoneme activity

**Figure 5:**
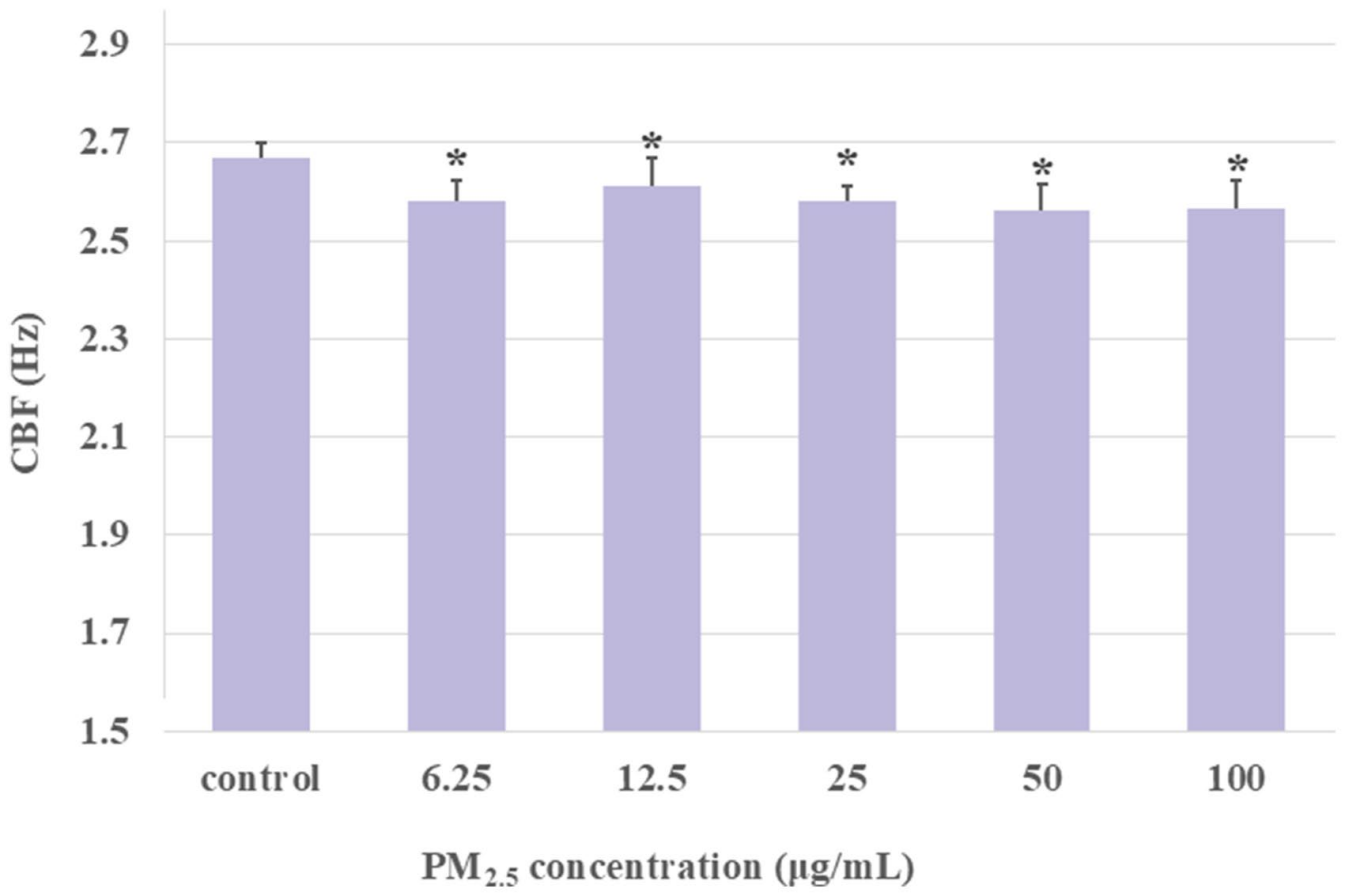
The CBF was detected after the ciliary axonemes had been exposed to different concentrations of PM_2.5_ (6.25, 12.5, 25, 50, 100 μg/mL) for 1 h. The CBF exhibited a decline over time in the context of prolonged PM_2.5_ stimulation. The prolonged exposure of the ciliary axonemes to PM_2.5_ resulted in a lower CBF in the PM_2.5_-exposed group than in the control group. Data were presented as mean ± S.D. of three independent experiments performed in triplicate. *p < 0.05, PM_2.5_ treated group compared to the control group.

The above study demonstrated that PM_2.5_ has a dual effect on CBF. Short-term exposure simulations of 10 min revealed a significant increase in CBF at low PM_2.5_ concentrations, contrasted with a marked decrease in CBF at higher concentrations. 50 μg/mL PM_2.5_ was found to significantly suppress CBF in ciliary axonemes. To further verify the changes in axonemes following prolonged exposure to PM_2.5_, extracted axonemes were treated with different concentrations of PM_2.5_ (6.25, 12.5, 25, 50, and 100 μg/mL) for 1 h, and the changes in CBF were measured every 30 s during the last 10 min of the stimulation. The experimental groups were as set as: control group, PM_2.5_-treated (6.25, 12.5, 25, 50, and 100 μg/mL) group. As illustrated in Figure 5, the mean CBF in all concentration groups of the PM_2.5_-treated (6.25, 12.5, 25, 50, and 100 μg/mL) group was lower than that of the control group (*p* < 0.05). Furthermore, even low concentrations of PM_2.5_ (12.5, 25 μg/mL) did not produce the same level of activation when stimulating axonemes as that observed in the previously simulated short-term exposure. These results suggested that long-term exposure to PM_2.5_ might result in a reduction in the activity of ciliary axonemes and a decrease in the beat frequency.

### The effect of cAMP on axonemes long-term exposed to PM_2.5_

**Figure 6:**
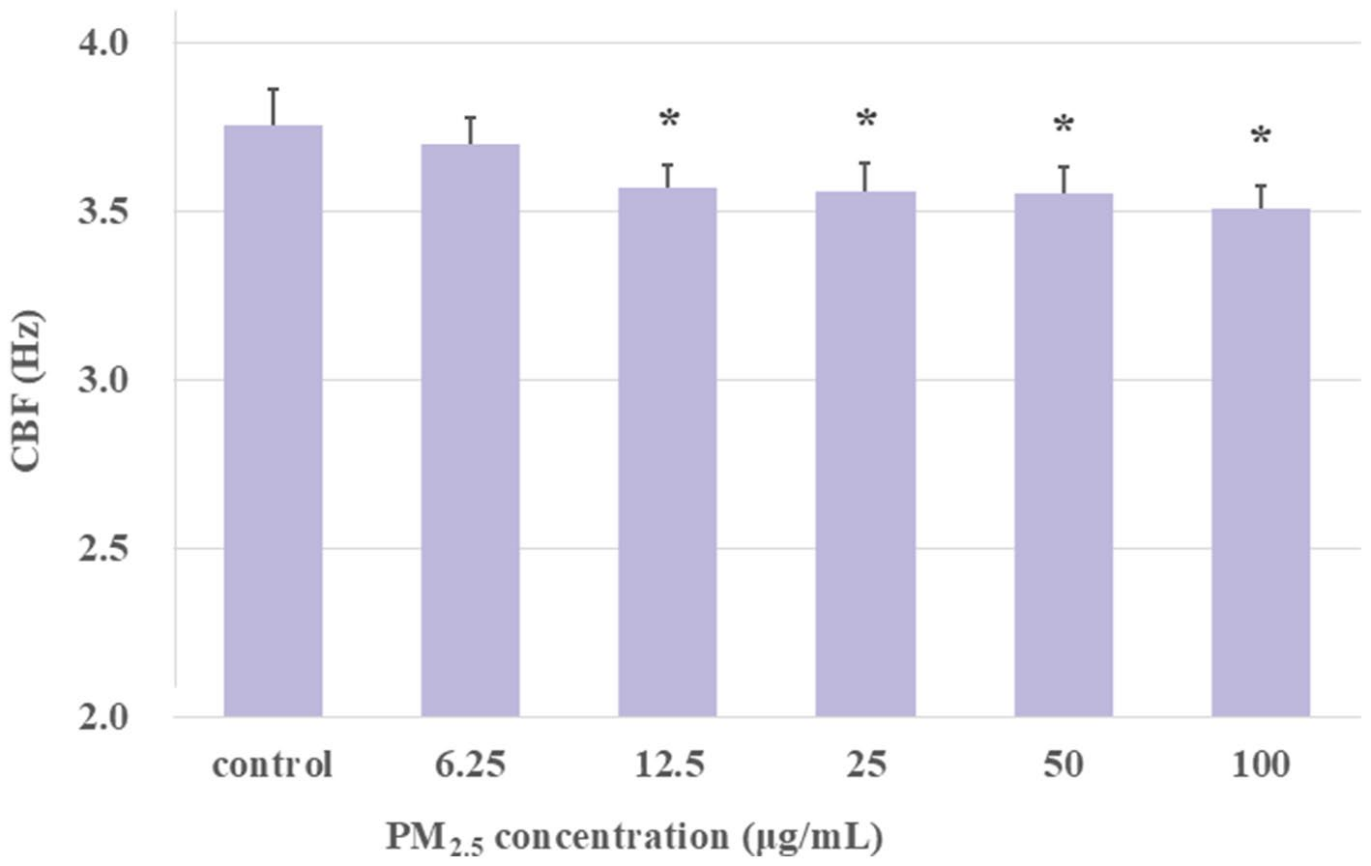
Exposure to PM_2.5_ for 1 h desensitized axonemes to cAMP stimulation. The results showed that under long-term exposure to PM_2.5_ at concentrations of 12.5 μg/mL and above, cAMP was unable to activate CBF to the same level as the control group. *p < 0.05, PM_2.5_-treated group compared with control group.

The aforementioned findings demonstrated that 1-h exposure to PM_2.5_ resulted in a decline in CBF. To further investigate the effect of long-term exposure to PM_2.5_ on the CBF of ciliary axonemes activated by cAMP, the extracted axonemes were treated with PM_2.5_ at concentrations of 6.25, 12.5, 25, 50, and 100 μg/mL for 1 h. At the last 10 min of the 1-h treatment, 10 μM cAMP was added, and CBF was measured every 30 s. Ciliary axonemes treated with cAMP alone (no PM_2.5_ exposure) were designated as the control group. The experimental groups were set as: control group, PM_2.5_ (6.25, 12.5, 25, 50, and 100 μg/mL) plus cAMP-treated group. As shown in Figure 6, the mean of CBF in the PM_2.5_ (6.25, 12.5, 25, 50, and 100 μg/mL) plus cAMP-treated group was markedly lower than that of the control group (*p* < 0.05). The findings demonstrated that, although the addition of cAMP activated the axonemes, Long-term exposure to PM_2.5_ impaired the ability of airway epithelial axonemes to exhibit cAMP-stimulated increases in CBF.

## Discussion

Air pollution has consistently been a global concern for human health, with numerous studies demonstrating that exposure to air pollution increased the incidence of lung diseases and mortality rates (31–33), particularly PM_2.5_ in the air (34). PM_2.5_ has been recognized as a major air pollutant posing serious public health risks. The World Health Organization (WHO) in their 2021 publication of the WHO Global Air Quality Guidelines revealed a reduction in the annual mean concentration of PM_2.5_ in the air quality guidelines (AQG level) from 10 μg/m^3^ in 2005 to 5 μg/m^3^. Even at lower exposure levels, PM_2.5_ could have adverse effects on health. Mucociliary clearance acts as the primary line of defense mechanism of the host respiratory tract, with ciliary beating representing a significant factor in its functionality. In this study, we explored the mechanism of PM_2.5_ on airway ciliary axon beating, which contributed to further understanding of PM_2.5_-induced respiratory diseases.

Firstly, we investigated the impact of varying concentrations of PM_2.5_ on axonal motility to elucidate the effects of PM_2.5_ on axonal CBF and the underlying mechanism. Initially, we measured the CBF of ciliary axonemes exposed to different concentrations of PM_2.5_ for 10 min. The findings indicated that CBF increased at low PM_2.5_ concentrations but decreased at high concentrations (Figure 2). At PM_2.5_ concentrations of 12.5, and 25 μg/mL, ciliary activity was significantly higher than that of the control group; however, when the dose was increased to 100 μg/mL, CBF was apparently lower than that of the control group. As known, after foreign compounds enter the respiratory tract, they adhere to the cilia of airway epithelial cells, which regularly beat to clear inhaled substances and produce mucus, a process known as MCC system. MCC serves as the primary innate defense mechanism in the lungs, with the normal functioning of cilia being essential for effective MCC. The frequency and amplitude of ciliary beating governed the velocity of MCC (35). Nevertheless, the potential impact of PM_2.5_ on the ciliary activity of airway epithelial cells has not been well described. Our results revealed that after exposure to low concentrations of PM_2.5_, ciliary axonemes immediately increased the CBF of airway epithelium. To minimize the mechanical damage of PM_2.5_ to airway epithelium, the body promptly activated the MCC, clearing invasive particles from the airway as quickly as possible and accelerating particle transport to the airway, thereby increasing CBF (36). It is reasonable to suggest that airway epithelial cells have the ability to immediately promote ciliary activity upon PM_2.5_ inhalation, which accelerates the initial host defense of clearing particles from the airway. Additionally, the study has shown that in mucus-free murine and human airway epithelia, cilia increased their beat frequency to maintain particle transport (37). However, when the PM_2.5_ concentration reached 100 μg/mL, the beating frequency of ciliary axonemes decreased significantly, indicating that high concentration of PM_2.5_ might induce axoneme damage, thereby reducing axonal motility. A study using a chronic rhinitis rabbit model found that PM_2.5_ exposure severely damaged cilia and cilial structures, and even induced irreversible mucosal remodeling (21). As a component of MCC, cilium axonemes constitute the body’s first line of defense against PM_2.5_. When the concentration of PM_2.5_ became too high, the axonemes might succumb to damage, reducing their ability to beat effectively.

The mechanism by which PM_2.5_ regulated CBF was further investigated in the following study. The most common mechanisms known to increase CBF included calcium-dependent NO/cGMP-dependent phosphorylation, cAMP-dependent phosphorylation, and Ca^2+^ action (38). PKA, composed of two regulatory subunits and two catalytic subunits, undergoes dissociation upon binding of cAMP to its regulatory subunits, releasing the catalytic subunits to phosphorylate downstream substrates (39). Numerous studies have evidenced the pivotal function of the cAMP-PKA signaling pathway in the maintenance of cellular homeostasis and oxidative metabolism. Therefore, to explore whether PM_2.5_ affected CBF through the cAMP-PKA pathway, we introduced PKA activators (cAMP) and PKA inhibitors (PKI) in this study. The results shown in Figures 3, 4 demonstrated that PKI effectively inhibited the stimulatory effect of low-concentration PM_2.5_ on CBF and that PM_2.5_ significantly reduced the activity of cAMP-treated axonemes. The above results indicated that the cAMP-PKA pathway indeed participated in the PM_2.5_-induced changes in CBF. Additionally, it was reported that an increase in calcium ion concentration activated calcium-sensitive adenylate cyclase (tmAC), catalyzing the conversion of ATP to cAMP, resulting in increased cAMP production and ultimately leading to an increase in CBF, enhancing the ability of airway epithelial cells to clear mucus and foreign particles (40). The intracellular cAMP level appeared to be an important determinant of the transport function of pulmonary mucociliary clearance (41). In summary, cAMP signaling enhanced ciliary activity, thereby accelerating MCC, and the cAMP-PKA signaling pathway played a key role in initiating the host defense response in airway epithelial cells. Overall, our findings revealed that PM_2.5_ affected the activity of airway epithelial cilia by acting on the cAMP-PKA signaling pathway.

In addition to the changes in PM_2.5_ concentrations affecting toxic effects, exposure time was also identified as a crucial influencing factor. Therefore, in this study, we sought to simulate the long-term, low-dose PM_2.5_ exposure that humans experience in their living environment, and to measure the impact of different concentrations of PM_2.5_ exposure for 1 h on ciliary activity. The results indicated that, unlike previous short-term exposure studies, in the extended period, CBF was significantly decreased in all PM_2.5_-exposed groups compared to the control group (Figure 5). This suggested that long-term PM_2.5_ exposure significantly inhibited ciliary activity, raising the question of whether there was potential damage to the structure and function of the cilia. Previous research has shown that the beating of respiratory epithelial cilia was a vital component of the mucociliary transport apparatus (42–44). Structurally, cilia are microtubule-based organelles that extend from the basal body, which is a centriole located at the cell apex and contains the axoneme. The axoneme is the microtubule cytoskeleton of the cilium, comprising nine doublet microtubules encircling a central pair (9 + 2). Each doublet microtubule has an inward-directed dynein arm and an outward-directed dynein arm, which generate the force required for movement in an ATP-dependent process (45). Changes in CBF depend on the outer dynein arms, hence, defects in the outer dynein arms are associated with decreased CBF (46–50). Additionally, studies have found that exposure to silica particles could cause abnormal ultrastructure of respiratory cilia, including central microtubule absence, microtubule disarray, and axoneme loss, as well as a notable reduction in the number of ciliary axonemes and basal bodies in the ciliated epithelium, with the proportion of abnormal axonemes increasing with exposure concentration (51). Therefore, the decrease in CBF under long-term PM_2.5_ exposure might have been due to structural damage to the ciliary axonemes by PM_2.5_, particularly to the outer dynein arms. Additionally, we tested the effect of adding cAMP to see if it could counteract the decrease in CBF caused by prolonged PM_2.5_ exposure. The findings demonstrated that, even with the addition of cAMP, different concentrations of PM_2.5_ exposure still resulted in CBF levels below the control group (Figure 6). This suggested that long-term PM_2.5_ exposure downregulated the cAMP-PKA pathway involved in axoneme beating. Previous studies have shown that the phosphorylation level of PKA in rat hippocampal neurons was significantly inhibited after prolonged PM_2.5_ exposure (52). However, the specific potential mechanisms by which PM_2.5_ inhibited the cAMP-PKA pathway required further investigation. The results of the impact of long-term PM_2.5_ exposure on CBF demonstrated that prolonged exposure to PM_2.5_ led to a significant decrease in CBF, potentially due to structural damage and downregulation of the cAMP-PKA pathway, highlighting the need for further research into the underlying mechanisms.

## Conclusion

**Figure 7:**
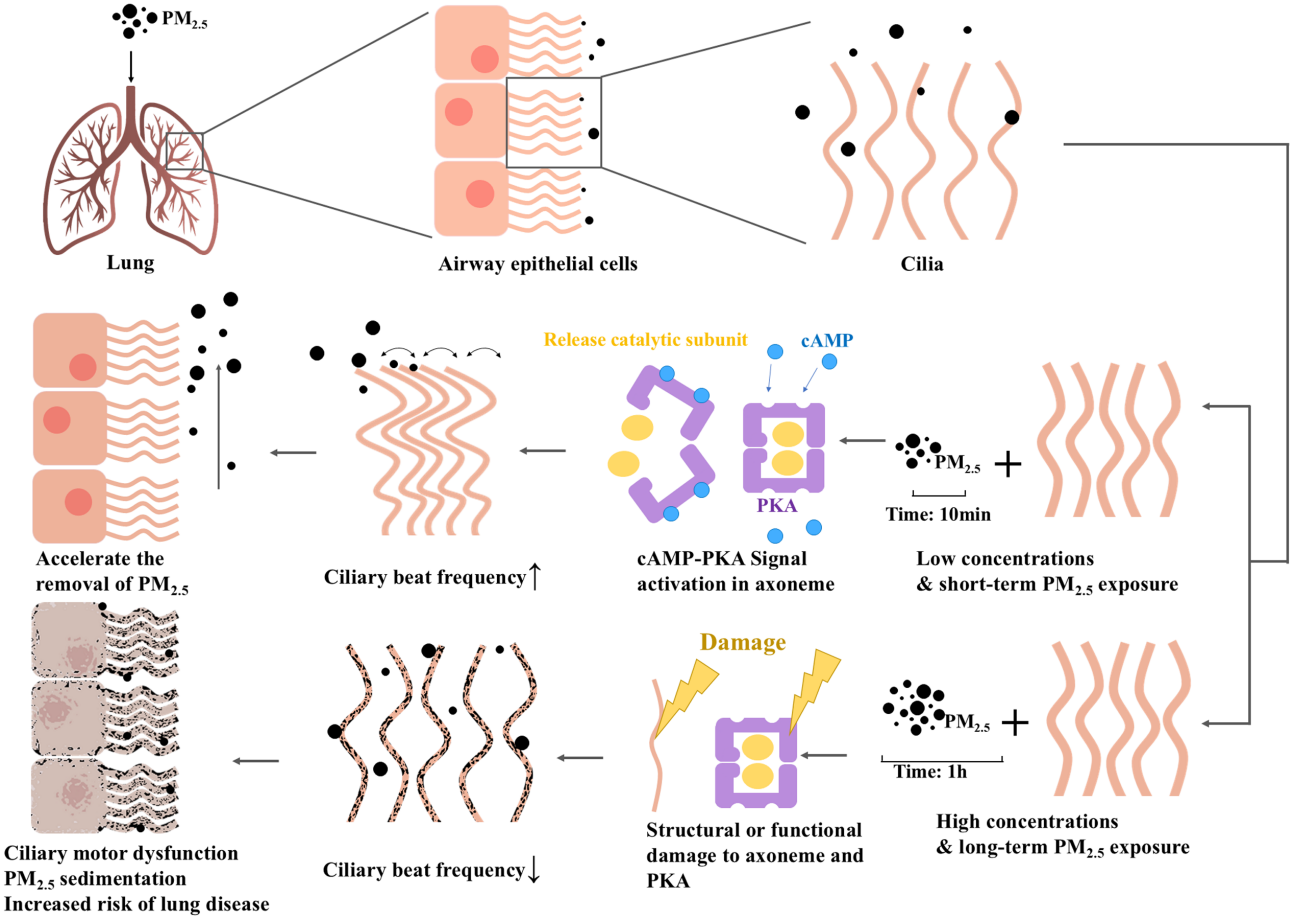
Schematic illustration of the effect of PM2.5 exposure on CBF in airway cilia. PM2.5 regulated ciliary activity via the cAMP-PKA signaling pathway, where short-term and low-concentration stimulation increased CBF, whereas long-term and high-concentration exposure reduced it.

The present study focused on early changes in ciliary activity under different concentrations and durations of PM_2.5_ exposure. Our data provided new findings that inhaled PM_2.5_ stimulated ciliary activity through the cAMP-PKA signaling pathway, and that short-term stimulation at low concentrations increased ciliary CBF, whereas high concentrations and prolonged exposure to PM_2.5_ reduced ciliary activity. This might be an important mechanism by which PM_2.5_ affected the airways from responding to both normal and pathological stimuli, and it was closely related to the development of lung diseases. The entire article is sorted out in Figure 7.

## Data Availability

The raw data supporting the conclusions of this article will be made available by the authors, without undue reservation.
